# Application of Domain- and Genotype-Specific Models to Infer Post-Transcriptional Regulation of Segmentation Gene Expression in *Drosophila*

**DOI:** 10.3390/life11111232

**Published:** 2021-11-13

**Authors:** Maria A. Duk, Vitaly V. Gursky, Maria G. Samsonova, Svetlana Yu. Surkova

**Affiliations:** 1 Mathematical Biology and Bioinformatics Laboratory, Peter the Great Saint Petersburg Polytechnic University, 195251 St. Petersburg, Russia; duk@mail.ioffe.ru (M.A.D.); m.samsonova@spbstu.ru (M.G.S.); 2 Theoretical Department, Ioffe Institute, 194021 St. Petersburg, Russia; gursky@math.ioffe.ru

**Keywords:** *Drosophila* embryo, segmentation genes, post-transcriptional regulation, gene expression, pattern formation, dynamical model, discrepancies between mRNA and protein levels

## Abstract

Unlike transcriptional regulation, the post-transcriptional mechanisms underlying zygotic segmentation gene expression in early *Drosophila* embryo have been insufficiently investigated. Condition-specific post-transcriptional regulation plays an important role in the development of many organisms. Our recent study revealed the domain- and genotype-specific differences between mRNA and the protein expression of *Drosophila hb*, *gt*, and *eve* genes in cleavage cycle 14A. Here, we use this dataset and the dynamic mathematical model to recapitulate protein expression from the corresponding mRNA patterns. The condition-specific nonuniformity in parameter values is further interpreted in terms of possible post-transcriptional modifications. For *hb* expression in wild-type embryos, our results predict the position-specific differences in protein production. The protein synthesis rate parameter is significantly higher in *hb* anterior domain compared to the posterior domain. The parameter sets describing Gt protein dynamics in wild-type embryos and *Kr* mutants are genotype-specific. The spatial discrepancy between *gt* mRNA and protein posterior expression in *Kr* mutants is well reproduced by the whole axis model, thus rejecting the involvement of post-transcriptional mechanisms. Our models fail to describe the full dynamics of *eve* expression, presumably due to its complex shape and the variable time delays between mRNA and protein patterns, which likely require a more complex model. Overall, our modeling approach enables the prediction of regulatory scenarios underlying the condition-specific differences between mRNA and protein expression in early embryo.

## 1. Introduction

Gene expression is controlled at the mRNA and protein levels. This control includes transcriptional regulation, mRNA processing, regulation of translation, protein stability and degradation. The exact contribution of regulation at the mRNA level versus regulation at the protein level is a subject of long-standing discussion [1,2,3,4,5].

Until recently, mRNA expression was considered to be the main determinant of protein expression. However, the analyses of whole-genome data in most biological systems revealed a low correlation between mRNA and protein levels [6,7,8,9,10,11]. On average, only 40% of the protein concentrations can be explained by the known values of the mRNA concentrations [1,12,13].

A low correlation between mRNA and protein levels was detected during the development of many model organisms [6,7,8,9,10,11]. For example, in the course of nematode development, correlation coefficients between mRNA and protein at four developmental stages drop from 0.41 to 0 [7]. Moreover, the concentrations of mRNA and protein may have different temporal dynamics [8,9].

The thorough analysis of regulatory interactions using dynamical models reveals the complex regulatory scenarios underlying the low protein–mRNA correlation. This approach can distinguish between proteins whose expression requires post-transcriptional regulation and those whose levels can be explained by mRNA concentration dynamics. A recent paired transcriptomic/proteomic study encompassing 14 timepoints during *Drosophila* embryogenesis (0–20 h of development) detected a relatively small mRNA–protein correlation. Interestingly, mathematical models explained 84% of protein time-courses based on the mRNA dynamics. The remaining protein–mRNA pairs were considered to be under complex post-transcriptional control [10].

*Drosophila* has a segmented body plan, which is laid down during the first three hours of development via the regulatory cascade of segmentation genes. Segmentation gene expression splits the major axis of an embryo into increasingly narrower domains corresponding to future parasegments. Maternal genes establish the anterior–posterior polarity of the embryo, zygotic gap genes are expressed in broad domains and zygotic pair-rule genes are expressed in narrow stripes encompassing about four cells [14,15,16,17,18]. Segmentation genes code for transcription factor proteins. Up to the end of cleavage cycle 14A, cellularization of the *Drosophila* embryo is not completed and gene interactions proceed through the diffusion of gene products between neighboring nuclei [19]. As the spatio-temporal dynamics of expression play the major role in the segmentation gene network, the preferable method of data acquisition is whole-mount staining in situ.

After the onset of zygotic transcription, pattern formation mechanisms in the segmentation system have been fully attributed to the regulation at the transcriptional level, and this network has served as a model system for transcriptional regulation for decades [15,16]. Consequently, in most cases, the previously published mathematical models of gap gene regulatory dynamics used protein patterns as a proxy for mRNA expression [20,21,22,23,24,25,26]. All these studies combined transcriptional and post-transcriptional regulation, implicitly stating the absence of post-transcriptional regulation in the embryo.

The spatial expression patterns of zygotic segmentation genes at the level of mRNA and protein are very similar at first glance. There are only few studies that quantitatively compare the mRNA and protein expression of segmentation genes. These studies revealed the following discrepancies between mRNA and protein expression of segmentation genes: (1) differences in the position of posterior borders of expression domains at the mRNA and protein levels [20,27] due to the temporal shifts caused by asymmetric transcriptional repression [20]; (2) different dynamics of mRNA and protein concentrations within gap gene posterior domains [28].

Remarkably, before maternal-to-zygotic transition in the *Drosophila* embryo, the protein gradients of key segmentation regulators such as maternal Cad and Hb are established through the mechanisms of translational repression and are classic examples of post-transcriptional regulation [29,30,31,32]. Thus, the absence of post-transcriptional regulation of the zygotic segmentation genes should be put to a rigorous test.

The first modeling study on post-transcriptional regulation in the *Drosophila* segmentation gene system was published by Becker et al. [28]. The authors applied mathematical modeling approach to the posterior ‘bell-shaped’ domains of three gap genes *Kruppel* (*Kr*), *knirps* (*kni*) and *giant* (*gt*) in wild-type embryos to infer whether their expression could be explained by the major parameters of protein production from mRNA, or some additional post-transcriptional regulation may be required. The modeling results showed that post-transcriptional regulation is not necessary for pattern formation in the system; however, it is necessary to maintain a proper protein concentration within each domain in early and late cleavage cycle 14A [28].

Our recent analysis revealed that the differences between segmentation gene expression at the level of mRNA and protein are domain- and genotype-specific. We found discrepancies between mRNA and protein expression of *gt*, *hunchback* (*hb*) and *even-skipped* (*eve*) in wild-type embryos and *Kr* mutants [33].

The variation between mRNA and protein levels in the particular tissue and developmental stage may be a consequence of post-transcriptional regulation. For example, a recent publication reported the complex mode of spatial and temporal post-transcriptional regulation of the receptor tyrosine kinase *tie1* mRNA in zebrafish embryo [34].

Here, we sought to analyze if post-transcriptional regulation plays any role in the domain- and genotype-specific discrepancies between mRNA and protein expression of *Drosophila* segmentation genes [33]. A number of studies reported differential changes in mRNA and protein expression in response to experimental perturbations [4,35,36]. In *Kr* null mutants, segmentation gene expression is significantly altered [26,37,38,39], so we used the mutation in the *Kr* gene as a perturbing factor for the segmentation system.

We apply the dynamic modeling approach suggested by Becker et al. [28] to infer whether the protein expression domains could be recapitulated from the corresponding mRNA expression [33]. We use a set of parameters describing basic processes such as protein synthesis, diffusion and degradation, as well as the delay in protein production from mRNA. The values of characteristic parameters are estimated by fitting the dynamic model to protein expression data for different conditions, namely, different genotypes and spatial positions along the main axis of the embryo.

If the model can describe the protein dynamics under these different conditions using the same set of parameter values, we will conclude that the observed difference between the mRNA and protein patterns is a consequence of the interplay between different rates and time scales of translation, degradation, and diffusion. On the contrary, if the condition-specific models provide an improvement in the description of protein dynamics and lead to parameter values varying across expression domains and/or genotypes, this will hint at the presence of some additional mechanisms required for spatial and/or genotype-specific variations in protein production.

If we fail to reproduce the dynamics of protein expression with both whole-axis and condition-specific models within a biologically plausible range of parameters, we do not interpret the modeling results. In our case, we failed to model the Eve protein pattern maturation characterized by the complex spatio-temporal dynamics [17,18,39].

For the first time, we use an in silico approach to infer the post-transcriptional regulation of the zygotic *hb* gene. As mentioned above, the maternal *hb* mRNA is post-transcriptionally regulated to form the anterior gradient of Hb protein at the early blastoderm stage [40,41]. Is the zygotic *hb* expression under post-transcriptional control, as in maternal *hb*?

A recent publication reported the translational regulation of a zygotic *hb* transcript, anteriorly expressed under the control of a proximal enhancer [42]. However, the role of post-transcriptional regulation in the formation of Hb endogenous pattern along the whole anterior–posterior (A–P) axis in cycle 14A still remains elusive. Here, we show that Hb protein production from mRNA requires domain-specific models. The value of protein synthesis rate parameter in the anterior domain is about two times higher compared to the posterior domain.

Unlike *hb*, *gt* expression in wild-type embryos is reproduced with a whole axis model. In *Kr* mutants, the domain-specific model is required for the anterior *gt* domain. However, the spatial discrepancy between *gt* mRNA and protein posterior expression domains in mutants [33] is described by the whole-axis model, thus rejecting the involvement of any additional post-transcriptional mechanisms.

Overall, in this paper, we infer post-transcriptional regulation within the segmentation gene system using the whole-axis and spatially restricted models for wild-type embryos and mutants.

## 2. Materials and Methods

### 2.1. Model and Data

We model the dynamics of the protein concentration $y_{i}$ in *i*th nucleus along the A–P axis of the *Drosophila* embryo as a result of translation from the mRNA with the concentration $u_{i}$, degradation, and diffusion between the neighboring nuclei, as follows [28]:

(1) $$\frac{dy_{i}\left(t\right)}{dt}=\alpha u_{i}\left(t-\tau \right)+D\left(n\right)\left[\left(y_{i-1}-y_{i}\right)+\left(y_{i+1}-y_{i}\right)\right]-\lambda y_{i},$$

where α is the protein synthesis constant, λ the degradation constant, D(n) the diffusion constant for the cleavage cycle *n*, and τ is the time delay required for the mRNA processing and protein translation.

**Figure 1:**
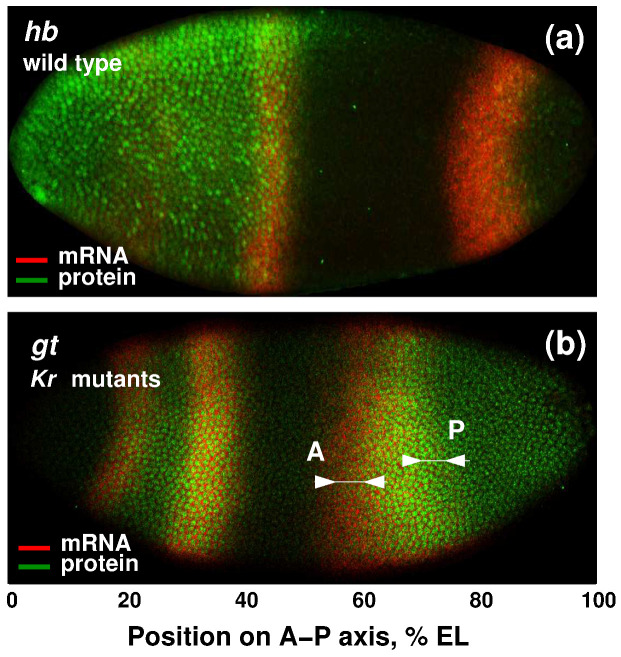
The examples of domain-specific discrepancies between protein and mRNA expression of gap genes *hb* and *gt*. (**a**) Image shows an individual wild-type embryo from mid-cycle 14A, stained for expression of *hb* mRNA and protein. mRNA expression declines across the anterior domain with a well-pronounced stripe at about 45% embryo length at the position of future parasegment 4 (PS4 stripe). On the contrary, anterior Hb protein expression retains high levels throughout cycle 14A. (**b**) *gt* posterior expression in *Kr* mutants is characterized by a significant shift in *gt* mRNA expression with respect to the Gt protein domain in the first part of cycle 14A. Arrows show the mismatch between the anterior (A) and posterior (P) border positions at mRNA and protein levels. Images were cropped and rotated to align the embryos (anterior to the left, dorsal is up). Modified from Figures 2 and 3 of [33]. Scale bar at the bottom of the figure indicates the positions of expression domains as percentage of embryo length. This scaling was applied to build the integrated data for model fitting. Grayscale images of separate confocal channels without preudocoloring are shown in Appendix A, Figure A1.

**Figure A1:**
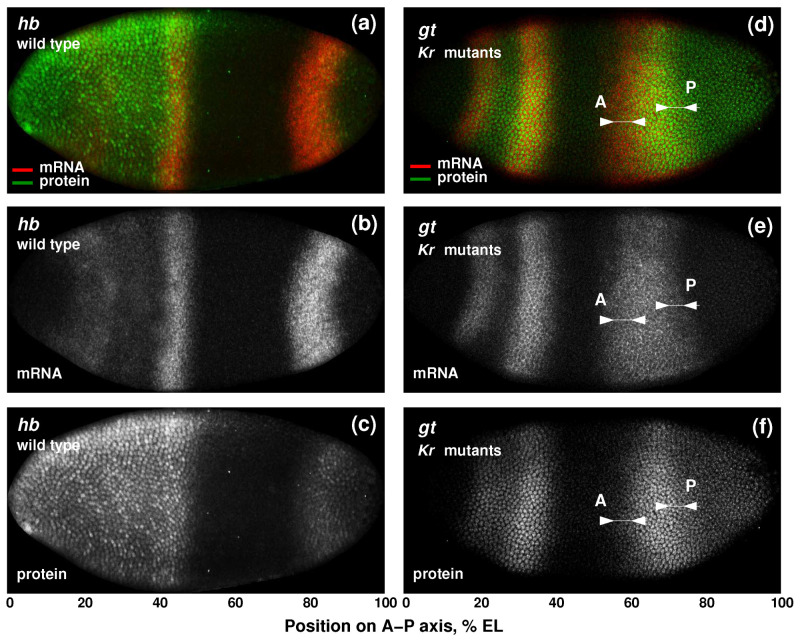
The examples of domain-specific discrepancies between protein and mRNA expression of gap genes *hb* and *gt*. (**a**) Image shows an individual wild type embryo from mid-cycle 14A stained for expression of *hb* mRNA and protein. (**d**) *gt* posterior expression in *Kr* mutants. Arrows show the mismatch between the anterior (A) and posterior (P) border positions at mRNA and protein levels. Grayscale images of separate confocal channels without preudocoloring are presented in panels (**b**,**c**) for *hb* and (**e**,**f**) for *gt*. Scale bar at the bottom of the figure indicates the positions of expression domains as percent of embryo length. See Figure 1 for the detailed legend.

The model was fitted to the previously published mRNA and protein expression data for *hb*, *gt* and *eve* [33]. Along with wild-type embryos (Oregon R stock), we considered *gt* and *eve* expression in *Kruppel* null mutants ($Kr^{-}$ embryos) from the *Kr${}^{1}$* amorphic allele [43]. Embryos were stained for mRNA expression using the Hybridization Chain Reaction (HCR) method, which includes hybridization and amplification steps [33]. This technique provides a high signal-to-background ratio, deep sample penetration and multiplexed mRNA imaging [44]. In a case of a combination of HCR and immunochemistry, the proteinase K treatment at the hybridization step was replaced by incubation with 80% acetone [45], see Supplementary protocol in [33]. After HCR, most embryos were stained with the primary antibodies against the protein products of segmentation genes, followed by incubation with the secondary antibodies conjugated to Alexa Fluors (Invitrogen) [18,33,39]. Finally, the embryonic nuclei were labeled with Hoechst 34580 DNA dye (Thermo Fisher) for the further extraction of quantitative data on gene expression [33]. The examples of confocal images of embryos that were double-stained for mRNA and protein expression are shown in Figure 1 and Appendix A, Figure A1. The discrepancies between the mRNA and protein patterns detected in experiments and used to interpret modeling results are described in the ‘Results’ section.

Gene expression patterns within cleavage cycle 14A were distributed into eight time classes of about 6.5 min each [18,33,39]. The quantitative data on gene expression have been processed and integrated (averaged) within each time class, as described earlier [46]. Here, we considered *gt* and *eve* integrated patterns without normalization on maximum expression to analyze the concentration dynamics of all expression domains along the A–P axis.

The mRNA and protein data values were interpolated in time using standard Matlab functions. The dynamics of all proteins except Hb were only considered during the cleavage cycle 14A (in 100 nuclei). For *hb*, the data for cycle 13 (in 50 nuclei) were also used, accounting for the mitosis event between the cycles. The schedule for the mitosis and its duration were adopted from [21]. Assuming a fast mRNA degradation and no transcription during the mitosis, we set mRNA concentration for *hb* to zero during the mitosis. As the distance between the nuclei is halved after the division, we have D(14)=4D(13) for *hb* [28].

### 2.2. Model Fitting and Hypotheses

We used several means of fitting the model to the data in accordance with the various considered hypotheses. Firstly, we fitted the model to the protein data on the whole A–P axis (‘whole-axis fits’), which corresponds to the assumption that the parameters controlling protein dynamics (the parameters α, λ, *D*, and τ) do not depend on the spatial position. According to this assumption, the processes of protein synthesis, degradation, and diffusion are uniform across the embryo. Secondly, if the whole-axis fits produce visible defects in the model solution, we refitted the model to the protein patterns within separate spatial domains localized at the anterior and posterior of the embryo (‘domain-specific fits’), with a subsequent comparison of the model quality in these domains between the domain-specific and whole-axis fits. If the domain-specific fits improve the fitting quality as compared to the whole-axis fits, we conclude that the protein dynamics in the embryo cannot be explained by the uniform parameters, and domain-specific parameters should be used. After that, we compare the optimized values of all parameters obtained in the whole-axis and domain-specific fits and find the parameters that demonstrate a significant difference between these fits. Such parameters, and processes associated with these parameters, are thus candidates for possible spatial dependence. This spatial dependence can be further investigated to be interpreted in terms of possible post-transcriptional modifications exhibiting themselves in this parametric nonuniformity. Finally, if the domain-specific fits do not improve the whole-axis fits, we conclude that the nonuniformity hypothesis can be rejected.

### 2.3. Parameter Optimization

The parameter values were estimated by minimizing the following quality functional, which describes the model solution’s deviation from the data:

(2) $$V=\sum_{i,j}\frac{{\left(y_{i}^{\mathrm{data}}\left(t_{j}\right)-y_{i}^{\mathrm{model}}\left(t_{j}\right)\right)}^{2}}{\sigma_{i}{\left(t_{j}\right)}^{2}},$$

where *i* and *j* enumerate the nuclei and time points, respectively, at which data are present, and $\sigma_{i}\left(t_{j}\right)$ is the standard deviation from data. For the analysis of domain-specific models, this functional can be split into parts associated with the proximity of data and model solutions in the anterior or posterior parts of the embryo related to the expression domains of a gene under consideration:

(3) $$V=V^{\mathrm{ant}}+V^{\mathrm{post}},$$

where $V^{\mathrm{ant}}$ and $V^{\mathrm{post}}$ are defined by (2) but the sum in that formula contains only nuclei *i* which appear in respectively the anterior or posterior expression domain.

The quality functional was minimized in Matlab using the simulated annealing method with a constraint of 1000 iterations. As a control, we performed an unconstrained minimization several times and concluded that the results did not much differ. To reduce possible overfitting, we performed multiple parameter optimization runs for each model setting and analyzed the results using an ensemble approach, i.e., accounting for all parameter values, not just those that provide the minimum value of the quality functional. The parameter values from multiple optimization runs were filtered by excluding sets with τ<1 and *V* values exceeding the 75th percentile of all optimized values. In the resulting filtered ensemble, we found the parameter values closest to the vector ($\hat{\alpha }$, $\hat{\lambda }$, $\hat{D}$, $\hat{\tau }$) consisting of the median values of each parameter, by minimizing the normalized Euclidean distance between this vector and parameters in the ensemble. The resulting parameter values represent the central trend in the optimization results.

We estimated the quality of fitting using two measures. As a quantitative measure, we considered the distribution of the quality functional values obtained from multiple optimization runs. In addition, we ensured that the peaks of the expression domains showed the correct dynamics, which underlies the previously reported difference between protein and mRNA dynamics [33].

## 3. Results

### 3.1. Position-Specific Models for Hunchback Expression in Wild Type Embryos

The dynamics of *hb* expression differ significantly at the mRNA and protein levels. Moreover, mRNA and protein expression show discrepancies, which vary with respect to spatial position along the A–P axis of an embryo [33].

In cleavage cycle 14A, *hb* is expressed in two domains in the anterior and posterior of the embryo and in the PS4 stripe at the position of future parasegment 4 [47,48]. The concentration of *hb* mRNA in the anterior domain significantly declines during cleavage cycle 14A, transforming into two weak stripes by gastrulation. However, the Hb protein retains high levels of expression (Figure 1a). The discrepancy between *hb* mRNA and protein levels in the anterior domain has been attributed to the slower rate of Hb protein degradation compared to mRNA [42,47].

Contrary to the anterior expression, *hb* mRNA concentration in the posterior domain rapidly increases during cycle 14A. Protein concentration in this domain increases slowly and reaches the mRNA level by the end of cycle 14A [33].

As a baseline model for *hb*, we performed multiple parameter optimization runs to fit the model (1) to the Hb protein pattern on the whole A–P axis (‘whole-axis fits’). This fitting experiment corresponds to the assumption that one set of parameter values can explain the protein dynamics in all embryo parts. To find out if the anterior and posterior Hb domains could be associated with different parameter values, we performed separate multiple optimization in each domain and verified that these fits could improve the quality of the solution within the domains as compared to the whole-axis fits.

**Figure 2:**
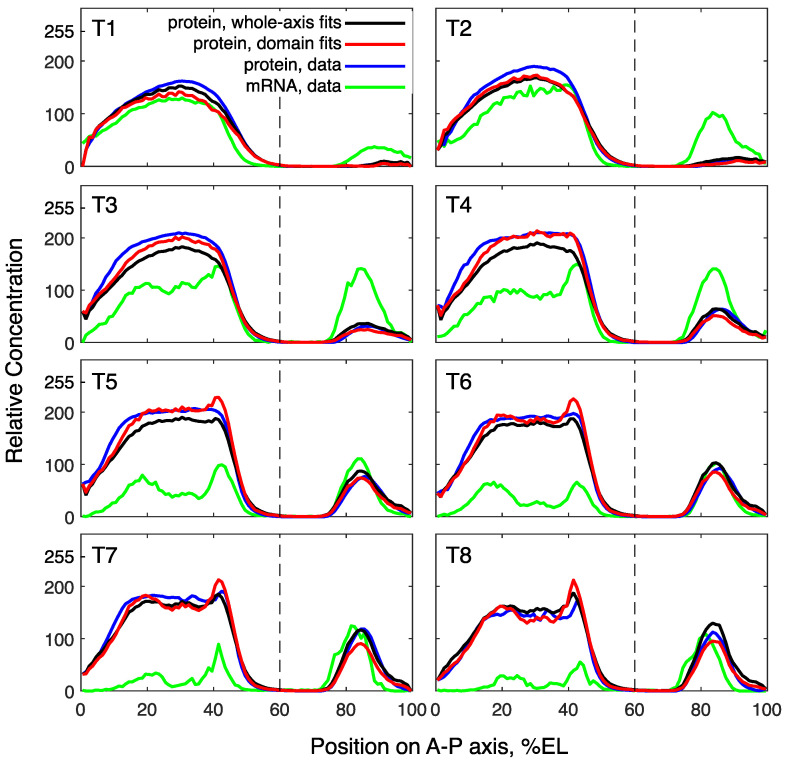
Model solutions for *hb* in comparison with the data. The solutions correspond to the best fits for the whole axis (black) and the domains (red), shown at eight time classes (T1–T8) in cleavage cycle 14A. The vertical dashed line separates the anterior and posterior *hb* domains at 60% embryo length (EL).

**Figure 3:**
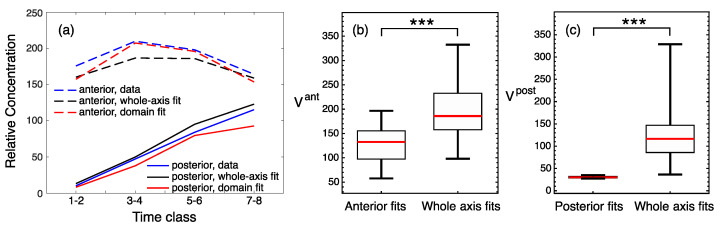
Quality measures for *hb* solutions from the whole-axis and domain-specific fits. (**a**) The dynamics of the maximal protein concentrations within the anterior (dashed lines) and posterior (solid lines) domains calculated from the data (blue), best whole-axis fit (black), and best domain-specific fits (red). The curves connect the concentration values averaged over two consecutive time classes. (**b**) Boxplots for values of the anterior-domain quality functional $V^{\mathrm{ant}}$ calculated for all parameter values from the two fitting experiments. Right, fits for the whole axis; left, fits for the anterior domain only. (**c**) Same as in (**b**), but for comparison between posterior-domain fits with whole-axis fits. Statistical significance of the difference between the medians according to the Mann–Whitney test: *p*-value < 0.001 (***).

The solutions corresponding to the best fits in these computational experiments match the data, with a visually good precision (Figure 2). A quantitative comparison of fit quality shows that the domain-specific models better describe the data (Figure 3). All models demonstrate good correspondence to the data in the dynamics of domain peaks (Figure 3a), reproducing qualitatively different dynamical regimes in the two domains, as observed in the data [33], but both domain-specific fits resulted in the peak dynamics with slightly better proximity to the data than the whole-axis fits. The parameter optimization within the domains led to significantly smaller median errors as compared to the whole-axis model (Figure 3b,c). Therefore, domain-specific fits indeed improved data description, so we may conclude that the anterior and posterior parts of the embryo can be associated with different parameters controlling Hb dynamics.

**Figure 4:**
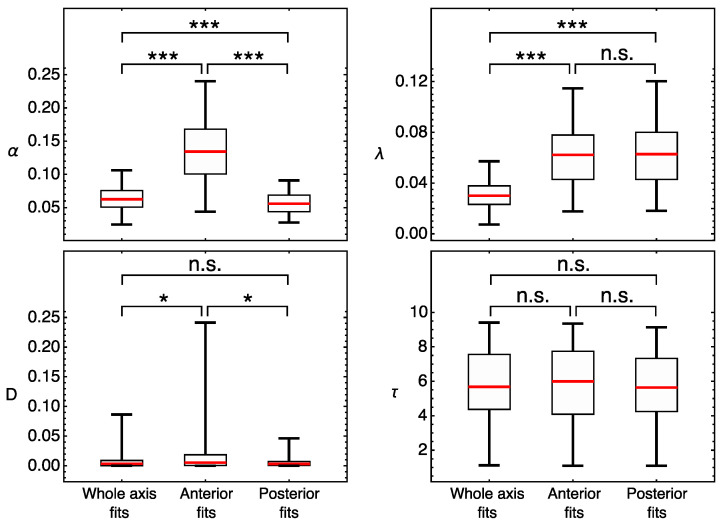
Distributions of parameter values obtained by multiple optimization runs in three fitting experiments in the model for Hb. The parameter values closest to the medians: (α, λ, *D*, τ) = (0.058, 0.027, 0.003, 6.11) for the whole axis, (0.131, 0.055, 0.005, 5.99) for the anterior domain, and (0.050, 0.052, 0.003, 5.50) for the posterior domain. Statistical significance of the difference between the medians according to the Mann–Whitney test: *p*-value ≥ 0.05 (not significant, n.s.), *p*-value < 0.05 (*), *p*-value < 0.001 (***).

To find the specific parameters responsible for this difference between domains, we compared the parameter values obtained in the domain-specific models. We apply the ensemble approach and use all sets of parameter values that resulted from the multiple optimization in this comparison, focusing on possible difference between the medians of parameter distributions. This analysis shows that the median degradation rate constant λ and the delay time τ are very similar in the anterior and posterior domain fits (Figure 4). The diffusion coefficients *D* have very small values in all Hb models. This is consistent with the earlier prediction that diffusion is not required for the correct formation of gap protein domains [20,24,28]. Thus, we do not consider the domain-specific difference in median *D* values.

In contrast to other parameters, the synthesis rate constant α in the posterior Hb domain is much smaller than that in the anterior domain (Figure 4). Different synthesis rate constants in the anterior and posterior domains correlate with the different relations between mRNA and protein patterns observed in the data (Figure 2). In contrast to the posterior domain, the mRNA in the anterior strongly decreases over time, while the protein is maintained at a high level. Through modeling, we found that this differentiation between the domains is due to α and not the other parameters, which could theoretically also participate in the effect. The latter cannot be obtained only on the basis of data analysis and without modeling.

### 3.2. Modeling Gt Protein Dynamics in Wild-Type Embryos and *Kruppel* Mutants

In cleavage cycle 14A, *gt* is expressed in the anterior and posterior of the embryo. *gt* anterior expression is rather complex: a small head domain at the anterior tip of the embryo is followed by the broad band, which is progressively spit into two stripes [18,37,49]. The ‘bell-shaped’ posterior domain shifts over time in the anterior direction due to the transcriptional repression by the other gap genes, which are more posteriorly expressed. As a result of this repression, *gt* posterior mRNA domain is located asymmetrically with respect to the Gt protein domain [20]. In *Kr* mutants, *gt* expression is decreased compared to wild-type embryos at both mRNA and protein levels [26,39]. The wide posterior domain is anteriorly displaced to the position of neighboring *kni* domain [38]. This is accompanied by the significant shift in *gt* mRNA domain with respect to Gt protein domain and results in the mismatch between the positions of domain borders at mRNA and protein levels (Figure 1b).

We were unable to find a set of parameter values that would provide a solution describing both wild-type and mutant expression data with good quality. Therefore, in what follows, we analyze the two genotypes separately.

**Figure 5:**
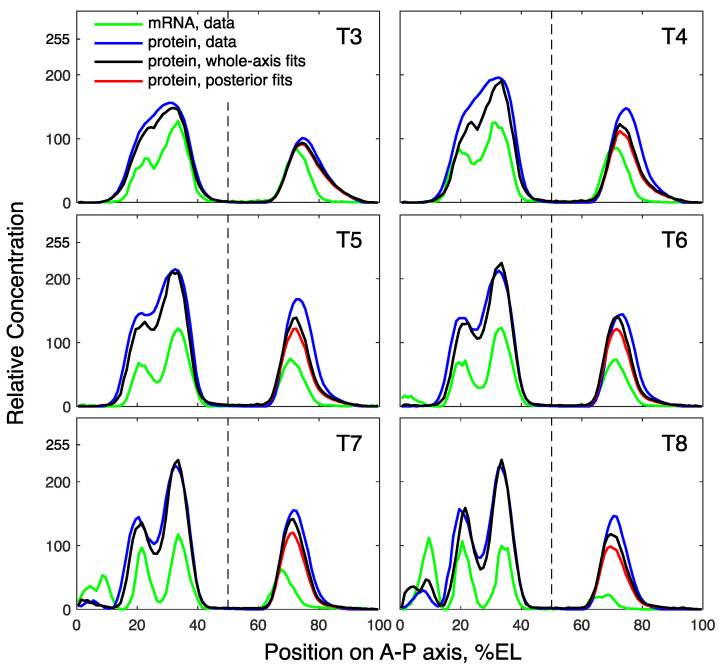
Model solutions for *gt* in comparison with data in wild-type. The solutions correspond to the best fits for the whole axis (black) and for the posterior Gt domain only (red), shown at six time classes (T3–T8) in cleavage cycle 14A. The vertical dashed line separates the anterior and posterior *gt* domains at 50% embryo length (EL).

**Figure 6:**
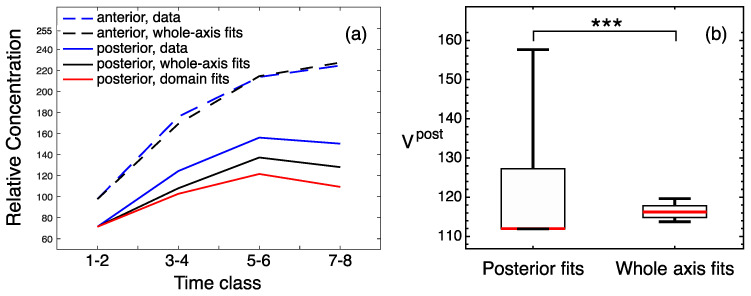
Quality measures for wild-type *gt* solutions in fittings for the whole axis and posterior domain. (**a**) The dynamics of maximal Gt protein concentrations within the anterior (dashed lines) and posterior (solid lines) domains calculated from the data (blue), the best whole-axis fit (black), and the best posterior-domain fit (red). The curves connect the concentration values averaged over two consecutive time classes. (**b**) The boxplots for values of the posterior-domain quality functional $V^{\mathrm{post}}$ calculated for all parameter values from the two fitting experiments. Right, fitting for the whole axis; left, fitting for the posterior domain. Statistical significance of the difference between the medians according to the Mann–Whitney test: *p*-value < 0.001 (***).

Multiple optimization for the wild-type data on the whole axis resulted in a solution that approximated the protein pattern in the anterior and posterior domains with different qualities, with some visible defects in the posterior part (black curve in Figure 5). The domain peaks in the solution and Gt expression data are very close to each other for the anterior domain, but not the posterior one (black and blue curves in Figure 6a). A possible explanation for this domain difference in the solution quality may be related to the fact that the anterior Gt pattern is wider and has a larger amplitude compared to the posterior one, and thus provides a larger input into the quality functional. The small defects in model solution during the division of anterior domain into two parts in time classes 4 and 5 are likely the consequence of the significant variability in shape observed in the data from individual embryos from this time period [18]. As a consequence, we did not perform separate optimization for the anterior domain and attributed the parameter values from the whole-axis fits as suitable for the anterior domain.

To find out if the posterior *gt* domain can be associated with different parameter values, we fitted the model to the posterior Gt pattern only and verified that these fits can improve the quality of the solution in this domain as compared to the whole-axis fits. A direct minimization of $V^{\mathrm{post}}$ resulted in very scattered values of diffusion constant *D* and production time τ, so that these parameters filled almost the entire search space, probably because the posterior Gt is too simple in shape. We fixed *D* and τ at their values from the best whole-axis fit and optimized only α and λ in the posterior-domain fitting. This optimization resulted in a slightly smaller median $V^{\mathrm{post}}$ compared to the whole-axis fits (Figure 6b), but did not lead to qualitative improvements (Figure 5 and Figure 6a). Following our fitting quality measures (see ‘Materials and methods’), we rejected the hypothesis about the spatial inhomogeneity of the parameters controlling Gt dynamics in wild-type embryos, since the domain-specific model does not provide an essential improvement compared to the model on the whole axis.

**Figure 7:**
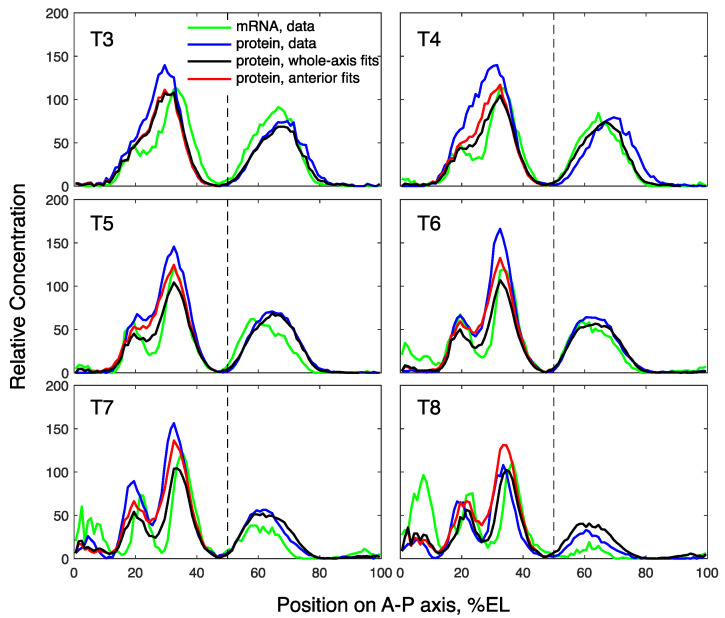
Model solutions for *gt* in comparison with data in *Kr* mutants. The solutions correspond to the best fit for the whole axis (black) and for the anterior *gt* domain only (red). The vertical dashed line separates the anterior and posterior *gt* domains at 50% embryo length (EL).

**Figure 8:**
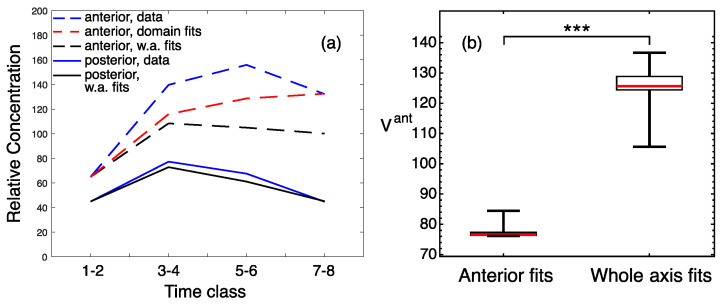
Quality measures for Gt solutions from the whole-axis and anterior-domain fits in $Kr^{-}$ embryos. (**a**) The dynamics of domain-specific maximal protein concentrations. (**b**) The boxplots for values of the anterior-domain quality functional $V^{\mathrm{ant}}$ calculated for all parameter values from the two fitting experiments. Statistical significance of the difference between the medians according to the Mann–Whitney test: *p*-value < 0.001 (***).

In contrast to the wild-type, the whole-axis fits in the $Kr^{-}$ embryos produced a solution that describes the posterior Gt domain better than the anterior one (black curve in Figure 7). The dynamics of the posterior expression peaks in the solution is in good correspondence with the data, while the anterior peaks exhibit an essential deviation from the data (Figure 8a). Therefore, we accepted the whole-axis fits as suitable for describing the posterior domain, but performed multiple optimization for the anterior domain.

The anterior-domain fits resulted in a solution that still shows visible deviations from the data in terms of amplitude, but these defects are smaller than those for the whole-axis fit (red curves in Figure 7 and Figure 8a). The anterior-domain fits demonstrate a significantly smaller error than the whole-axis fits (Figure 8b), thus quantitatively confirming the improvement in the anterior Gt. Therefore, these results indicate that the anterior Gt in $Kr^{-}$ embryos requires a separate domain-specific model, thus leading to the possibility of spatial inhomogeneity in parameters in the mutant.

**Figure 9:**
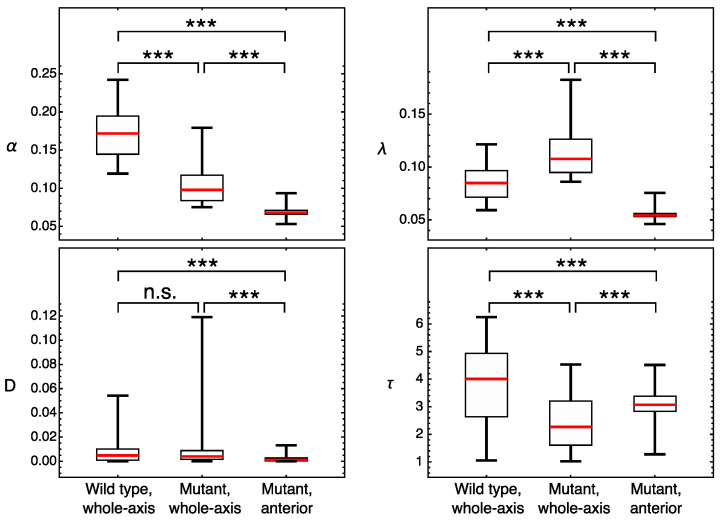
Distributions of parameter values obtained by multiple optimization in the model for Gt under different conditions. The parameter values closest to the medians: (α, λ, *D*, τ) = (0.174, 0.086, 0.005, 4.12) for the wild-type, whole-axis fits, (0.101, 0.110, 0.004, 2.16) for the mutant whole-axis fits, and (0.068, 0.054, 0.001, 3.09) for the mutant anterior-domain fits. Statistical significance of the difference between the medians according to the Mann–Whitney test: *p*-value ≥ 0.05 (not significant, n.s.), *p*-value < 0.001 (***).

Figure 9 shows how parameter values for Gt vary between domains and genotypes, accounting for the fact that parameters from the whole-axis fits in the mutant are attributed to the posterior Gt domain. The synthesis and degradation rate constants in the anterior part of the mutant embryo are, on average, smaller than those in the posterior, while the time delay τ is larger. Our failure in fitting the model to the joint data from the wild type and $Kr^{-}$ embryos indicates that there is no unified set of parameter values that are able to simultaneously describe the protein dynamics in these genotypes, thus suggesting that these parameters are genotype-specific. Comparing the average parameter values obtained by optimization in genotype-specific models (Figure 9), we see that the wild-type production rate constant α is significantly larger than those obtained for two domains in the mutant. This result correlates with the fact that the Gt expression levels in the mutant are approximately 1.5 times lower than those in the wild-type. The degradation constant λ in the wild type is between the mutant values for the anterior and posterior domains. The wild-type value of time delay τ is larger than in the mutant, but closer to the mutant value for the anterior domain. As in the case of Hb, the values of the diffusion coefficient *D* are very small in all conditions (Figure 9).

### 3.3. Model Fails to Reproduce Full Dynamics of Even-Skipped Expression

Unlike gap genes, the pair-rule gene *eve* is expressed in seven narrow stripes, which are formed in a variable sequence and rate during first three time-classes of cycle 14A [18]. In *Kr* mutants, *eve* expression dynamics are even more complex than in wild-type embryos: some stripes merge together while the others progressively divide [33,39].

Our experimental results revealed stripe-specific differences between the dynamics of *eve* mRNA and protein expression: (1) in early cycle 14A *eve*, mRNA concentration within stripe 7 is much higher then Eve protein concentration, but later mRNA and protein levels even out [33]; (2) the delays between pattern formation at the level of mRNA and protein vary from about 6.5 to 13 min depending on a time class [33]; (3) starting from mid-cycle 14A *eve*, mRNA pattern becomes more anteriorly distributed with respect to the Eve protein pattern due to the variable temporal shifts of posterior stripes [27,33].

**Figure 10:**
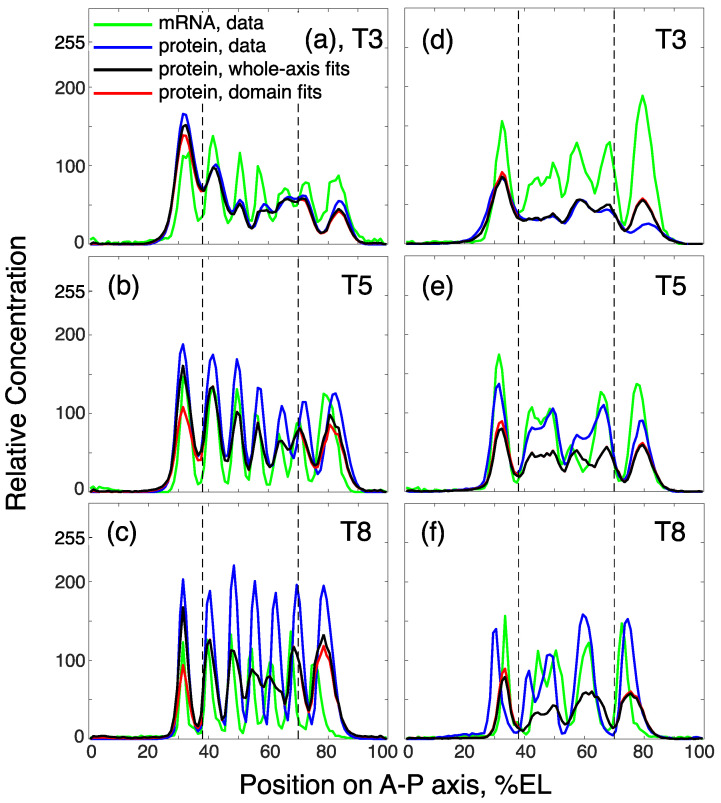
Model solutions for *eve* in comparison with data in (**a**–**c**) wild type and (**d**–**f**) $Kr^{-}$ embryos. The solutions correspond to the best fits for the whole axis (black) and the anterior and posterior domains (red), shown at three time classes (T3, T5, and T8) in cleavage cycle 14A. The vertical dashed lines separate the considered anterior and posterior *eve* domains at 0–38 and 70–100% embryo length (EL), respectively.

All these expression peculiarities impose inevitable constraints on the models’ ability to correctly reproduce Eve protein dynamics based on the mRNA dynamics. We obtained the solution as a result of the whole-axis fits that showed good correspondence to the wild-type and mutant data in time classes 3 (black curves in Figure 10a,d) and 4 (not shown). Later in cycle 14A, the solution provided a rather good approximation of stripe positions, but produced partially merged central stripes in wild-type embryos (Figure 10b,c). The major problem was the model solution’s failure to reproduce stripe amplitudes starting from time class 5 (Figure 10b,c,e,f).

**Figure A2:**
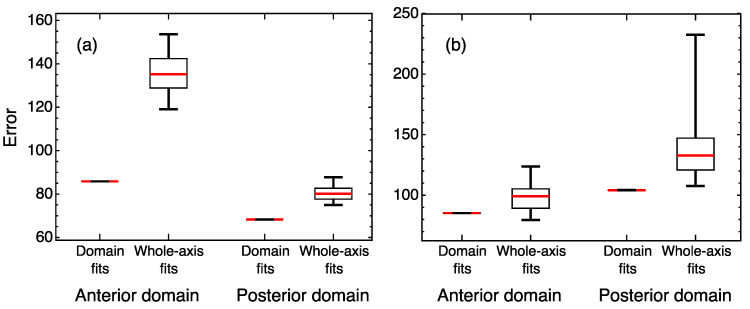
Errors in various fitting experiments for the Eve data in (**a**) wild type and (**b**) $Kr^{-}$. The distributions of $V^{\mathrm{ant}}$ and $V^{\mathrm{post}}$ are shown obtained in multiple optimization runs in the anterior and posterior domains, respectively, in comparison with the values calculated for parameter values from the whole-axis fits.

Next, we tried to fit the model to individual stripes within the *eve* pattern. We separated the anterior and posterior domains associated with the predominant expression in the first and last stripe, respectively (dashed lines in Figure 10), and the model was separately refitted to the Eve pattern within each domain. As in the case of similar fitting experiments for Gt, keeping all parameters free resulted in an almost uniform distribution of the optimized parameter values in the search space, probably because the pattern within the domains was too simple. We solved this problem by fixing parameters *D* and τ at the best-fit values from the whole-axis fits, and thus optimizing only α and λ. The domain fits did not provide a better approximation of domain peaks compared with the whole-axis fits in late cycle 14A (red curves in Figure 10), but showed a smaller error due to the improved fitting in the anterior and posterior tails of the pattern (Appendix A, Figure A2).

**Figure A3:**
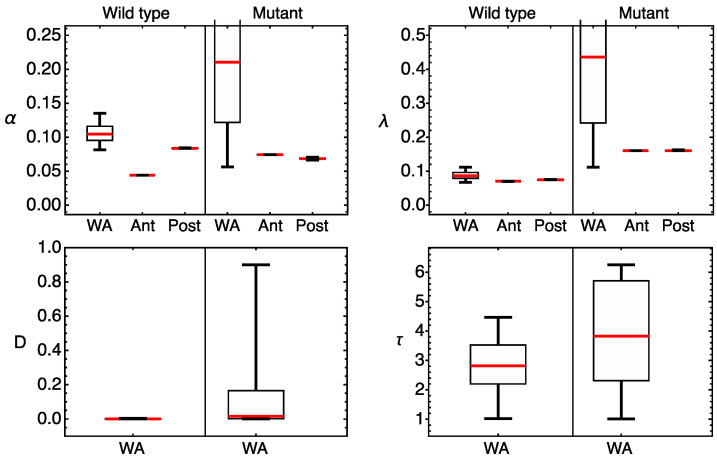
Distributions of parameter values obtained by multiple optimization in the model for Eve under different conditions. WA, fits on the whole axis; Ant, fits in the anterior domain; Post, fits in the posterior domain. The box-plot for the whole-axis fits in the mutant is cropped for better visibility in relation to other box-plots.

Despite the good approximation in early time classes, the total inability of the model to reproduce *eve* expression levels in mid–late cycle 14A rejects the possibility of considering model parameters for further analysis (Appendix A, Figure A3). As similar approximation defects are observed in both wild-type embryos and *Kr* mutants, we conclude that the *eve* expression dynamics are too complex to be reproduced by the rather simple model utilized in this study [28]. The formation of *eve* seven stripes through a series of complex shapes, with variable time delays between mRNA and protein pattern development at different timepoints [33], likely requires a more sophisticated approach than is necessary for a case of gap genes. Contrary to the large amounts of experimental data on the transcriptional regulation of pair-rule genes, the translatability of stripe-specific transcripts in space and time during embryogenesis has been much less investigated. Future experiments on the molecular mechanisms of translational dynamics are required to extend the model to accurately reproduce Eve protein expression.

## 4. Discussion

The segmentation gene system in *Drosophila* integrates the genes coding for transcription factors and sculpting the future body plan of a fruit fly in first three hours of development. This system has been used as a model of transcriptional regulation for decades [50,51].

Post-transcriptional regulation has been well studied for the two maternal effect genes lying upstream of the zygotic segmentation network—*caudal* and *hb* [52]. However, much less is known about the spatio-temporal dynamics of zygotic mRNA translation and the involvement of post-transcriptional mechanisms in the formation of zygotic protein patterns, including zygotic Hb.

Condition-specific post-transcriptional mechanisms play an important role in the development of many organisms. Post-transcriptional regulation may be tissue-specific [34,53,54,55], or induced by environmental or internal perturbations [36,56].

In this paper, we used mathematical modeling to test whether the production of segmentation proteins in the early *Drosophila* embryo is condition-dependent, i.e., varies with respect to the spatial position of the expression domain or due to mutations. The model describes the time-delayed linear synthesis of protein from mRNA, as well as protein degradation and diffusion [28]. If these model parameters do not change depending on the A–P position of segmentation gene expression domain, we consider that no additional post-transcriptional regulation is required for patterning. If such changes exist and the position-specific approach improves the modeling solution, we hypothesize some additional regulation of protein production.

Our approach allows predictions to be made regarding the involvement of condition-specific post-transcriptional regulation in segmentation gene pattern formation, and these predictions can be further considered as the hypotheses for experimental design. Below we discuss model predictions for each specific gene and condition.

### 4.1. Model Predicts Position-Specific Regulation of Hb Protein Production

Our results predict the position-specific regulation of Hb protein production. The two *hb* expression domains show different values of the synthesis constant α, which is more than two times higher in the anterior domain compared to the posterior domain. However, the degradation constant λ, characterizing protein half-life, is nearly equal in both domains (Figure 4).

In the earlier publications, it was suggested that high levels of anterior Hb protein are exclusively maintained due to the slow protein degradation rate [42,47]. Since our model for Hb describes the synthesis of this protein starting from cleavage cycle 13, the excess of the anterior Hb due to the synthesis prior to cycle 13 could potentially influence the observed difference in α values between the domains. In our models, the values of the degradation rate constant λ correspond to a zygotic Hb protein half-life of approximately 15–25 min, which is longer than in Gt and the other gap proteins [28]. Given the length of cleavage cycles 13 and 14A is about 16 and 50 min, respectively, it is unlikely that any significant amounts of Hb protein synthesized earlier than cycle 13 persist in the mid-cycle 14A embryo, thus ruling out the excess of long-lived Hb in the anterior domain as an exclusive explanation for the domain difference in α. According to Pultz et al., traces of maternal Hb protein can also persist in the anterior half of the *Drosophila* embryo until cycle 14A, but these levels are negligible compared to zygotic Hb expression at this stage [57].

Hb protein in the anterior domain is produced from the transcripts, which are regulated by proximal, distal and stripe enhancer elements [42,48,58]. Interestingly, a recent experimental assay of zygotic *hb* mRNA translation with a single-molecular resolution revealed that *hb* mRNA expressed under the control of a proximal enhancer is translationally repressed in the center of the anterior domain in early cycle 14A [42]. In cycle 13 *hb*, translation was uniform accross the anterior domain [42].

Since the translatability of *hb* transcripts regulated by other enhancers has not yet been studied, we can assume that the regulation of their translation could compensate for Hb protein synthesis in the anterior domain in cycle 14A. The Bicoid-dependent distal enhancer regulates *hb* transcription in parallel with the proximal enhancer, and the Hb protein synthesized from these transcripts is expressed in the anterior half of the embryo, except the anterior tip [58]. The stripe enhancer is activated in cycle 14A and this results in Hb protein expression in the posterior domain and in the PS4 stripe at about 45%EL [42,47,48]. Despite being synthesized from the mRNA produced at the same time, the PS4 stripe and posterior domain show considerable differences in Hb protein intensity (Figure 2), as captured by our models.

The position-specific difference in α, detected by our models, may reflect the upregulation of protein synthesis in the anterior, as well as the downregulation of protein production in the posterior of the embryo. Prior to maternal-to-zygotic transition, an anterior gradient of maternal Hb protein is formed via the translational repression of *hb* transcripts in the posterior half of the embryo [29,31]. This mechanism may potentially function in the later embryo and result in the reduced production of zygotic Hb in the posterior domain. However, it is unknown whether the maternal Nanos (Nos) protein, as well as the other components of the Nos-responsive element (NRE) complex [31], are present in cycle 14A. Nevertheless, translational repression by zygotic Nos has been intensely studied at later stages of *Drosophila* development [59,60,61].

Experimental evidence on Hb protein regulation in both the anterior and posterior parts of the embryo points towards the existence of position- and stage-specific post-transcriptional regulation of this gene. Further experiments, including the analysis of translation dynamics of *hb* mRNA produced under the control of different enhancers, are necessary to clarify the mechanisms of position-specific regulation of Hb protein synthesis predicted by our models.

### 4.2. Genotype-Specific Modeling of Gt Protein Dynamics

*gt* mRNA and protein domains show different expression dynamics in wild-type embryos and *Kr* mutants. The most intriguing issue is the displacement of the mRNA posterior domain relative to the protein domain in mutants (Figure 1b). The different spatial positions of the mRNA and protein expression domains of a gene may be a consequence of spatial post-transcriptional regulation. Mathematical modeling allows the existence of condition-specific variability in protein production to be predicted, and then considers this prediction as a hypothesis for the experimental design.

In our in silico experiments, we failed to find the unified parameter set describing Gt protein dynamics in wild-type embryos and mutants. This suggests that these parameter sets are genotype-specific.

For *gt* expression in wild-type, our results reject the domain-specific model of protein production, thus favoring the hypothesis of the absence of any position-specific post-transcriptional mechanisms. The whole-axis model solution reproduces the shape and positional dynamics of the posterior Gt domain. In accordance with Becker et al., we detected a small discrepancy in the maximum expression levels of posterior Gt domain between the solution and data, which does not affect overall concentration dynamics (blue and black solid lines in Figure 5 and Figure 6a). Remarkably, in the previous study, the solution approximation defects were detected within the posterior domains of three gap genes (*gt*, *Kr* and *kni*) in wild-type embryos from early and late cycle 14A. It has been suggested that such defects in model fitting could point to the requirement of additional post-transcriptional regulation for the maintenance of proper gap protein levels [28]. Our results do not fully support this conclusion, as the model well approximated the posterior expression of *hb* in wild-type embryos and *gt* in *Kr* mutants within the considered time intervals.

Interestingly, in *Kr* mutants, the posterior *gt* expression is perfectly reproduced by the whole-axis model (Figure 7). This rejects the involvement of post-transcriptional mechanisms in the spatial discrepancy between *gt* mRNA and protein posterior expression in mutants (Figure 1b). By contrast, the anterior *gt* domain fits improve the quality of the whole axis fits (Figure 7 and Figure 8). This spatial parameter inhomegeneity suggests that the mutation might induce some position-specific, post-transcriptional response.

Recently, a powerful system SunTag, developed to image the translation at single mRNA resolution in tissue culture cells [62,63], has been adapted to the *Drosophila* embryo [42,64]. The successful application of this system for the analysis of *hb* and *twist* mRNA translation suggests that this method might be adapted for other genes. As *twist* and *hb* both demonstrated spatial heteroheneity in mRNA translation efficiency, although caused by different mechanisms [42,64], the examination of *gt* translation in wild-type embryos and mutants will be of special interest in terms of genotype-specific model predictions.

### 4.3. Dynamic Sculpting of Eve Pattern: Modeling Failures and Perspectives

The mathematical model applied in this study provides a good approximation to analyze spatial parameter inhomogeneity for gap gene expression [28]. Here, we present a first attempt to model the protein concentration dynamics of the pair-rule gene *eve* based on its mRNA dynamics. Both whole-axis and domain-specific models reproduce early *eve* expression before stripe maturation (Figure 10a,d); however, in the second half of the cleavage cycle 14A, the model solution for most stripes is two times lower than the concentration of Eve protein (Figure 10b,c,e,f). This is observed in both wild-type embryos and *Kr* mutants. As the *eve* models fail to reproduce the previously reported expression dynamics [33], we do not analyze the resulting parameter values in terms of domain- and genotype-specific expression.

Contrary to gap domains, *eve* stripe formation is characterized by complex dynamics with variable time delays between pattern formation at the level of mRNA and protein during cycle 14A [33]. The presumptive temporal regulation of time delay can lead to the inability of our models to reproduce Eve protein expression starting from time class 5.

Besides the assumed delays for protein synthesis, *eve* pattern formation is accompanied by the shifts in more posterior stripes in the anterior direction over time [18,39]. The shift values differ between *eve* mRNA and protein patterns and lead to more anterior mRNA distribution with respect to protein [27,33].

To date, the detailed quantitative comparison of *eve* mRNA and protein expression within cycle 14A has only been reported for fixed embryos [33]. An investigation of *eve* stripe formation dynamics at both mRNA [65] and protein levels in living embryos will enable the creation of a more sophisticated model for post-transcriptional regulation that can truly reproduce the concentration levels of mature stripes.

### 4.4. Limitations of the Modeling Approach

The model (1) has several limitations, which were partially described in previous studies [28]. We can regard this model as one of the simplest possible ways of describing translation given the data we used, since it is based on linear differential equations that do not implement any specific post-transcriptional regulation mechanism. Making such implementations would involve non-linear generalizations, but their applicability to the biological object under study is unclear, and thus prone to generating new, unnecessary degrees of freedom (for example, new free parameters) [66]. On the other hand, even simple kinetic synthesis–degradation models are sufficient to reproduce the experimentally observed rich variety of possible relations between the dynamics of mRNA and protein concentrations [10]. Therefore, the modeling formalism that we used seems reasonable for the general questions we ask in the study, but would require more complex equations when testing specific post-regulation mechanisms.

We assume in the model that a single time delay parameter τ represents the duration of several complex biological processes, including mRNA synthesis on a gene, mRNA processing, its transportation to ribosomes, and participation in protein synthesis. It is highly likely that the duration of these processes demonstrates both temporal and spatial variation in the embryo. New experimental methods emerge that, in combination with modeling, provide important data on the processes, for example, allowing for the estimation of various parameters of the transcription cycle [67]. However, we still lack the necessary information to properly extend the constant τ to biologically reliable alternatives in the form of a function of time and space. Likewise, the constant values of the rate constants α and λ remain rough approximations for representing the underlying processes.

Another limitation concerns the fact that we do not know the actual mRNA and protein concentrations in the data and assume that those concentrations are proportional to the signal intensity. As we do not know the proportionality constant, it is implicitly included in the value of α, which we find from the data by solving the inverse problem. Multiplying $u_{i}$ by a constant *C* in the model Equations (1) can be compensated by dividing α by the same constant, and, similarly, the replacement of $y_{i}$ by $Cy_{i}$ in these equations can alternatively be performed by replacing α with Cα. Therefore, the unknown constant of proportionality between the signal in the data and the actual concentrations should theoretically be compensated for by the freedom in α.

**Figure A4:**
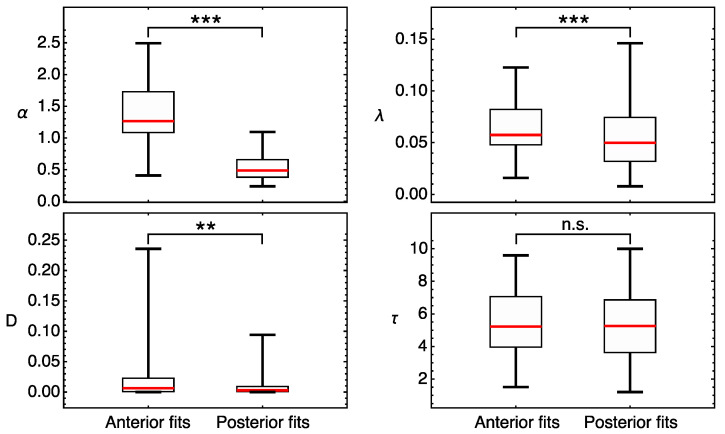
Distributions of parameter values obtained by multiple optimization runs in domain-specific fitting experiments in the model for Hb with the mRNA concentrations $u_{i}$ in the data replaced by $0.1u_{i}$. Statistical significance of the difference between the medians according to the Mann–Whitney test: *p*-value ≥ 0.05 (not significant, n.s.), *p*-value < 0.01 (**), *p*-value < 0.001 (***).

To check the reliability of our modeling results in the presence of this uncertainty, we performed test calculations to reproduce the difference between the anterior and posterior fits for Hb that were reported in Figure 4, but under the assumption that the mRNA concentrations are ten times smaller in the data. Thus, we refitted the model for Hb in the anterior and posterior domains, taking $0.1u_{i}$ instead of $u_{i}$ in the data. The resulting values of α are scaled by ten (to compensate for the decrease in mRNA concentrations, as expected) and are essentially smaller in the posterior domain, while τ does not show a significant difference between the domains (Figure A4). In contrast with Figure 4, the posterior-fit values of λ are different to the anterior-fit ones (Figure A4), but the difference is essentially smaller than that for α, so we may conclude that these test results demonstrate a similar tendency to that seen in the original calculations.

Finally, we note that our modeling results provide expression patterns that are very close to the data in positions and less accurate in amplitudes, confirming the previous conclusions [28]. Although we have shown that the uncertainty in the concentrations (and, thus, the expression pattern amplitudes) can be effectively incorporated in the parameter values, there is still a possibility that the amplitude differences that we observe in the modeling results under different conditions are not large enough to be biologically interpretable. More accurate data and models are required to clarify this possibility.

## Data Availability

Not applicable.
